# Advances in Sustainable Lutein Production: Sources, Technologies, and Functional Applications

**DOI:** 10.3390/foods15101717

**Published:** 2026-05-13

**Authors:** Setyo Budi Kurniawan, Suriya Vathi Subramaniam, Hassimi Abu Hasan, Muhammad Fauzul Imron

**Affiliations:** 1 Research Center for Environmental and Clean Technologies, National Research and Innovation Agency (BRIN), Jakarta Pusat 10340, Indonesia; setyo.budi.kurniawan@brin.go.id; 2 Department of Chemical and Process Engineering, Faculty of Engineering and Built Environment, Universiti Kebangsaan Malaysia, Bangi 43600, Selangor, Malaysia; p141373@siswa.ukm.edu.my (S.V.S.); hassimi@ukm.edu.my (H.A.H.); 3 Research Centre for Sustainable Process Technology (CESPRO), Faculty of Engineering and Built Environment, Universiti Kebangsaan Malaysia, Bangi 43600, Selangor, Malaysia; 4 Cleaner Production Research Group (CleanPro), Universiti Kebangsaan Malaysia, Bangi 43600, Selangor, Malaysia; 5 Study Program of Environmental Engineering, Department of Biology, Faculty of Science and Technology, Universitas Airlangga, Kampus C UNAIR, Jalan Mulyorejo, Surabaya 60115, Indonesia; 6 Research Group of Sustainable Environmental Systems and Infrastructure (SUSTAIN), Faculty of Science and Technology, Universitas Airlangga, Kampus C UNAIR, Jalan Mulyorejo, Surabaya 60115, Indonesia; 7 Department of Water Management, Faculty of Civil Engineering and Geosciences, Delft University of Technology, Stevinweg 1, 2628 CN Delft, The Netherlands

**Keywords:** agro-industrial byproducts, bioavailability, circular bioeconomy, functional foods, green extraction, microalgae, xanthophyll carotenoids

## Abstract

Lutein is a xanthophyll carotenoid widely recognized for its roles in eye health, antioxidant and neuroprotective effects, and the prevention of oxidative stress-related disorders. The growing demand for functional foods and nutraceuticals has heightened industry interest in sustainable lutein production. However, conventional sources such as green vegetables and marigold flowers face several limitations, including low bioavailability, seasonal variability, land-intensive cultivation, and sustainability concerns. Therefore, this review provides an updated, comprehensive, and integrated overview of sustainable lutein production, extraction technologies, and functional applications. This review discusses conventional dietary sources alongside emerging alternative platforms, including microalgae, agro-industrial byproducts, and bioengineered fermentation systems. Recent advances in green extraction technologies, particularly supercritical CO_2_, ultrasound-assisted, and enzyme-assisted extraction, are also critically evaluated due to their potential to improve extraction efficiency while reducing environmental impact. In addition, the applications of lutein in functional foods, nutraceuticals, and pharmaceutical products are also highlighted. This review further examines key technical challenges, including low bioavailability, high production and downstream processing costs, compound instability, extraction inefficiencies, lack of standardization, and scalability limitations. Future progress will depend on integrating circular bioeconomy strategies, artificial intelligence (AI)-assisted process optimization, sustainable biorefinery concepts, and advanced stabilization technologies to support economically viable and environmentally sustainable lutein production systems.

## 1. Introduction

Lutein is a naturally occurring pigment belonging to the xanthophyll subclass of carotenoids [1], a group of oxygenated carotenoids widely distributed in plants, algae, and certain microorganisms. Structurally, lutein is characterized by a C_40_ isoprenoid backbone with hydroxyl functional groups [2], which confer both its polarity and its biological functionality. Unlike provitamin A carotenoids, lutein does not convert into vitamin A but plays distinct physiological roles, particularly in ocular and systemic health [3]. Due to its lipophilic nature, lutein is typically associated with lipid-rich environments in foods and biological membranes, influencing its absorption and bioavailability in humans [4].

One of the most well-established roles of lutein is in eye health [5], where it accumulates selectively in the macula of the human retina, forming part of the macular pigment along with its isomer zeaxanthin [6]. This pigment acts as a natural blue light filter, protecting retinal tissues from photo-oxidative damage induced by high-energy visible light [7]. Additionally, lutein contributes to visual performance by improving contrast sensitivity and reducing glare [8]. Epidemiological and clinical studies have consistently associated higher dietary lutein intake with a reduced risk of age-related macular degeneration (AMD) and cataracts [9], two leading causes of vision impairment globally.

Beyond ocular benefits, lutein exhibits strong antioxidant activity, enabling it to neutralize reactive oxygen species (ROS) and mitigate oxidative stress at the cellular level [10]. This property is linked to broader health benefits, including anti-inflammatory effects [11], cardiovascular protection [12], and potential roles in cognitive function [13]. Its antioxidant capacity is particularly important in preventing lipid peroxidation in cell membranes [14], thereby maintaining cellular integrity and function.

Lutein has attracted considerable industrial and commercial interest due to its diverse applications in food, nutraceutical, pharmaceutical, cosmetic, and animal feed sectors [15,16]. In the food industry, lutein is widely used as a natural colorant and functional ingredient in dairy products, beverages, bakery products, infant formulas, and dietary supplements [4]. In nutraceutical and pharmaceutical applications, lutein is primarily marketed for eye health, particularly to reduce the risk of age-related macular degeneration (AMD), cataracts, and retinal oxidative damage [17]. It is also used in animal feed to enhance pigmentation in poultry and aquaculture products [18]. Commercially, lutein is incorporated into numerous eye-health supplements and functional products worldwide. For example, Kemin Industries produces FloraGLO^®^ Lutein, one of the most widely used lutein ingredients in dietary supplements and fortified foods. Similarly, DSM-Firmenich markets lutein-containing nutritional formulations for eye health applications (Vibelly™ Lutein), while BASF Nutrition supplies carotenoid-based ingredients for food and nutraceutical industries (Xangold^®^ natural lutein esters). Several commercial eye-health products, such as formulations from Bausch + Lomb (Ocuvite^®^ Lutein and PreserVision AREDS 2), also contain lutein in combination with zeaxanthin and antioxidant vitamins.

Despite this expanding demand, current production systems remain largely dependent on conventional plant-based sources [4], which are constrained by low bioavailability, seasonal variability, and resource-intensive cultivation. Limited understanding of lutein bioavailability across different food matrices [4], lack of standardized methodologies for extraction and quantification [18], high production and processing costs associated with alternative sources such as microalgae [19], and challenges related to lutein stability during processing and storage [20] are several critical research gaps that persist. Furthermore, the integration of sustainable production systems, such as the use of agro-industrial byproducts and bioengineered platforms [21,22], remains underexplored at an industrial scale.

Although numerous review articles on lutein have been published, most have focused primarily on its biological functions [23,24], nutritional significance [25,26], or individual production methods [19,27]. In contrast, relatively few studies have presented a comprehensive perspective that integrates sustainable production systems, extraction technologies, industrial applications, and emerging technological advancements within a single cohesive framework. Therefore, this review aims to provide a comprehensive and up-to-date overview of lutein, encompassing its chemical characteristics, bioavailability, biological functions, and conventional dietary sources, with particular emphasis on emerging and sustainable alternatives. In addition, this work critically evaluates current extraction and recovery technologies, discusses applications in food and health sectors, and identifies key challenges and future research directions. By synthesizing recent advances and highlighting existing gaps, this review aims to support the development of efficient, scalable, and sustainable lutein production systems that align with current industrial and environmental demands.

## 2. Chemistry and Bioavailability of Lutein

**Figure 1:**
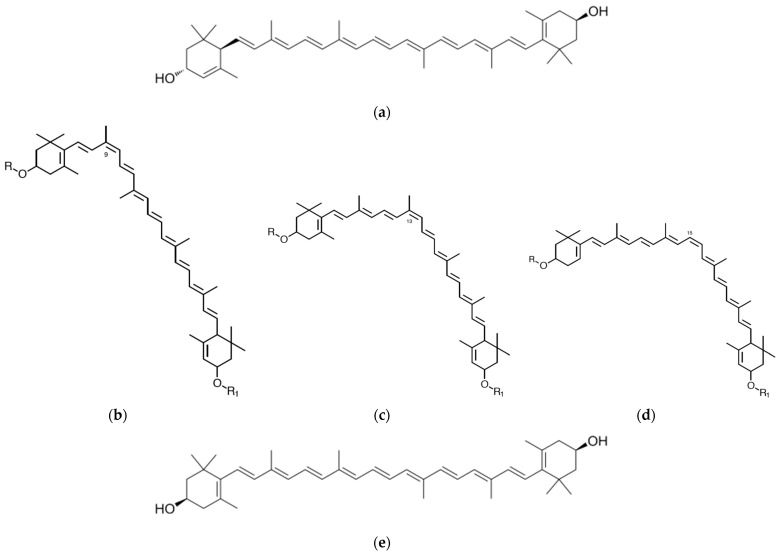
Molecular Structure of Lutein ((**a**). All-trans lutein, (**b**). 9-cis lutein, (**c**). 13-cis lutein, (**d**). 15-cis lutein, and (**e**). Zeaxanthin) [30].

Lutein (C_40_H_56_O_2_) is a member of the xanthophyll family of carotenoids [12], characterized by its oxygenated structure and polar functional groups. Chemically, it consists of a C_40_ tetraterpenoid backbone formed by eight isoprene units, with two hydroxyl (–OH) groups located at each end of the molecule [2] (Figure 1). These hydroxyl groups distinguish lutein from non-oxygenated carotenoids (carotenes) and contribute to its amphiphilic nature [28], enabling interactions with both lipid and aqueous environments in biological systems. The molecule contains a series of conjugated double bonds, which are responsible for its yellow coloration and its ability to quench singlet oxygen and scavenge free radicals [29].

### 2.1. Structure and Isomers

Lutein exists in several geometric isomeric forms, primarily due to the presence of double bonds that can undergo cis–trans isomerization [31]. The most biologically relevant and naturally abundant form is all-trans lutein (Figure 1a), which exhibits greater stability and bioavailability than its cis counterparts (9-cis, 13-cis, and 15-cis lutein) (Figure 1b–d) [4]. However, during food processing, storage, or exposure to heat and light, partial conversion to cis-isomers may occur [31]. These isomers often show reduced bioefficiency but may differ in their absorption and tissue distribution [32]. Lutein is also structurally similar to its isomer zeaxanthin (Figure 1e), differing only in the position of one double bond [33], yet both play complementary roles in human health, particularly in the retina.

### 2.2. Lipid Solubility

As a lipophilic compound, lutein is insoluble in water but readily soluble in lipids and organic solvents [34]. This property strongly influences the digestion, absorption, and transport of nutrients in the human body. In the gastrointestinal tract, lutein must first be released from the food matrix and incorporated into lipid droplets, then solubilized into mixed micelles composed of bile salts, phospholipids, and dietary lipids [4]. These micelles facilitate the transport of lutein across the intestinal epithelium. Once absorbed, lutein is incorporated into chylomicrons and transported via the lymphatic system into systemic circulation [17], where it is distributed to various tissues, including the retina.

### 2.3. Factors Affecting Absorption

#### 2.3.1. Dietary Fat

The presence of dietary fat plays a critical and well-documented role in enhancing lutein absorption due to its lipophilic nature [35]. As a fat-soluble carotenoid, lutein requires incorporation into lipid phases during digestion to become bioaccessible. The ingestion of dietary lipids stimulates the secretion of bile salts and pancreatic enzymes, which facilitate the formation of mixed micelles, colloidal structures composed of bile salts, phospholipids, fatty acids, and monoacylglycerols [36]. These micelles are essential for solubilizing lutein and transporting it across the unstirred water layer to the intestinal epithelium for absorption [37].

The efficiency of this process is strongly influenced by both the quantity and type of dietary fat. Moderate fat intake has been shown to significantly improve lutein bioavailability, while very low-fat meals can limit micelle formation and reduce absorption efficiency [38]. Additionally, the fatty acid composition is important; unsaturated fats (e.g., those from vegetable oils, nuts, and avocados) are generally more effective in promoting carotenoid absorption than saturated fats [39]. Empirical studies have consistently demonstrated that co-consuming lutein-rich foods with fat-containing meals, such as adding oil to salads, consuming vegetables with nuts, or pairing them with dairy products, can markedly increase plasma lutein levels [40]. This highlights the importance of considering not only the lutein content of foods but also the dietary context in which they are consumed.

#### 2.3.2. Food Matrix

The food matrix plays a decisive role in determining the bioaccessibility and subsequent bioavailability of lutein during digestion. Beyond its concentration, the physical and biochemical environment in which lutein is embedded governs how efficiently it is released, solubilized, and absorbed in the gastrointestinal tract [17]. In plant-based sources, particularly green leafy vegetables, lutein is tightly localized within chloroplasts and bound to pigment–protein complexes associated with photosynthetic membranes [41]. These structures are further protected by rigid cell walls composed of cellulose, hemicellulose, and pectin [42], which hinder the release of lutein during digestion. As a result, a significant fraction of lutein may remain inaccessible unless the matrix is disrupted through mechanical or thermal processing.

In contrast, animal-derived matrices such as egg yolk provide a highly favorable environment for lutein absorption [43]. In this case, lutein is naturally incorporated into a lipid-rich, emulsified system, which facilitates its direct incorporation into mixed micelles during digestion. This results in superior bioavailability, even though the absolute lutein content in egg yolk is lower than that in many plant sources.

#### 2.3.3. Processing

Food processing techniques can both enhance and degrade lutein bioavailability [4]. Mechanical processing and thermal treatments (such as cooking) can disrupt plant cell walls, facilitating lutein release and improving its accessibility [44]. However, excessive heat, light exposure, and oxidation may lead to lutein degradation or isomerization, reducing its biological activity [45]. Emerging processing technologies, such as encapsulation and emulsification, are increasingly explored to improve lutein stability and delivery in functional foods [46].

## 3. Biological Functions of Lutein

**Figure 2:**
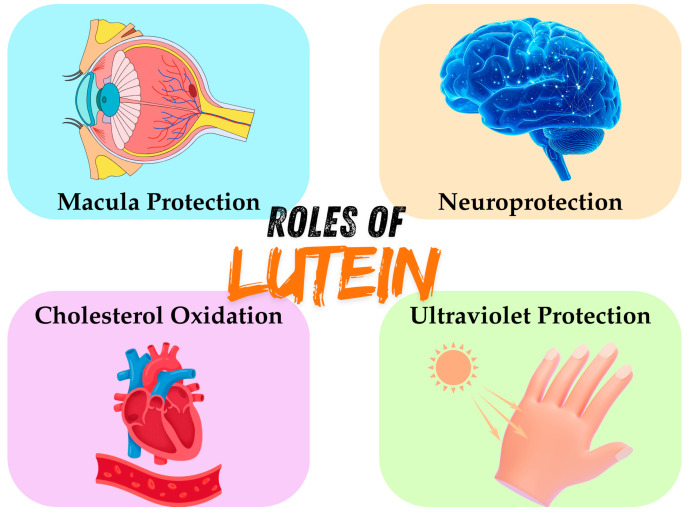
Biological Roles of Lutein in the Human Body.

Lutein is a multifunctional bioactive compound with diverse physiological roles in the human body, primarily attributed to its antioxidant capacity [47], light-filtering properties [48], and interaction with cellular membranes [49]. Unlike essential nutrients that serve as cofactors or structural components, lutein exerts its effects through protective and regulatory mechanisms [12], contributing to both visual and systemic health. Biological roles of lutein include macula protection, neuroprotection, cholesterol oxidation, and ultraviolet protection (Figure 2).

### 3.1. Ocular Protection and Visual Function

One of the most well-established functions of lutein is its role in maintaining eye health, particularly within the macula of the retina [6]. In ocular tissues, lutein and its isomer, zeaxanthin, selectively accumulate in the macula, where they function as a blue-light (400–500 nm) filter and antioxidant protector [50]. Mechanistically, lutein absorbs high-energy blue light, reducing photo-oxidative stress and limiting the formation of ROS in retinal cells. In addition, lutein stabilizes retinal cell membranes and protects photoreceptor cells from lipid peroxidation [8], thereby helping prevent age-related macular degeneration (AMD) and other retinal disorders [51]. Lutein has also been associated with increased macular pigment optical density (MPOD), which improves visual performance and reduces glare sensitivity [8].

### 3.2. Antioxidant and Anti-Inflammatory Activity

Lutein exerts its biological activities through several interconnected molecular and cellular mechanisms, rather than solely through general antioxidant effects. One of its primary mechanisms involves the quenching of reactive oxygen species (ROS) and singlet oxygen generated during oxidative stress. Due to its highly conjugated polyene structure, lutein can efficiently neutralize free radicals by electron transfer and energy dissipation, thereby protecting cellular lipids, proteins, and DNA from oxidative damage [47]. This mechanism is particularly important in tissues exposed to high oxidative stress, such as the retina and skin.

The conjugated double-bond system in its structure allows lutein to stabilize unpaired electrons, thereby preventing oxidative damage to lipids, proteins, and DNA [52]. This anti-inflammatory activity of lutein is closely associated with the modulation of cellular signaling pathways [53]. Studies have shown that lutein can suppress the activation of nuclear factor-kappa B (NF-κB), a key transcription factor involved in inflammatory responses [12]. By inhibiting NF-κB signaling, lutein reduces the expression of pro-inflammatory mediators such as tumor necrosis factor-α (TNF-α), interleukin-6 (IL-6), and cyclooxygenase-2 (COX-2) [53]. Furthermore, lutein may regulate oxidative stress-mediated inflammation by enhancing endogenous antioxidant defense systems, including superoxide dismutase (SOD), catalase, and glutathione peroxidase activities [53]. These properties also help prevent chronic diseases associated with inflammation and oxidative damage, including cardiovascular disorders and metabolic syndromes.

### 3.3. Role in Cellular Membrane Stability

Due to its amphiphilic structure, lutein integrates into biological membranes, particularly within phospholipid bilayers [49]. It is preferentially oriented across the membrane, where it enhances structural stability and protects lipids from peroxidation. By stabilizing membrane integrity, lutein helps maintain proper cellular function, including membrane fluidity, permeability, and signal transduction [12]. This function is especially important in tissues exposed to high oxidative stress, such as the retina and brain.

### 3.4. Cognitive Function and Neuroprotection

Emerging evidence suggests that lutein plays a role in brain health [54] and cognitive performance [13]. Lutein is one of the predominant carotenoids found in the human brain, particularly in regions associated with memory and learning [55]. Its neuroprotective effects are attributed to its ability to reduce oxidative stress in neural tissues [56], exert anti-inflammatory activity [47], and enhance neural efficiency and communication [5]. Collectively, these mechanisms support the maintenance of brain function and integrity. Furthermore, studies have indicated that higher lutein levels are associated with improved cognitive performance, including enhanced memory, processing speed, and executive function, particularly in aging populations [13].

### 3.5. Cardiovascular and Systemic Health

Lutein also contributes to cardiovascular health by reducing oxidative stress and inflammation within vascular tissues [53]. It has been associated with decreased oxidation of low-density lipoprotein (LDL) cholesterol [57], improved endothelial function [58], and a reduced risk of atherosclerosis [59]. Additionally, lutein may play a role in metabolic regulation and immune function [12], although these areas require further investigation.

### 3.6. Skin Protection and Photoprotection

Lutein provides protective effects against ultraviolet (UV) [60] and blue light-induced skin damage [61]. By neutralizing free radicals generated by UV exposure, lutein helps prevent premature skin aging, including lipid peroxidation, collagen degradation, and inflammation [62]. Its role in skin health includes enhancing skin hydration and elasticity, reducing photo-induced oxidative damage, and supporting overall skin integrity [60].

## 4. Conventional Dietary Sources of Lutein

**Figure 3:**
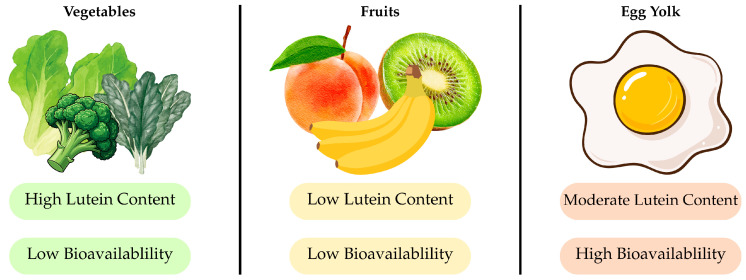
Conventional Dietary Sources of Lutein and Its Bioavailability.

Lutein is widely distributed in plant-based foods, particularly in green leafy vegetables, where it plays a role in photosynthesis and photoprotection. In human diets, conventional sources of lutein are primarily derived from vegetables, fruits, and certain animal-based products, especially egg yolk (Table 1). However, the concentration and bioavailability of lutein vary significantly depending on the source, food matrix, and preparation methods, as summarized in Figure 3 and detailed in the following subsections.

### 4.1. Green Vegetables

Green vegetables are considered the richest natural sources of lutein, with particularly high concentrations found in kale (14.7–39.6 mg/100 g) (one of the highest known dietary sources) [69]. Other vegetables such as spinach (6.26–8.95 mg/100 g), cilantro (7.70 mg/100 g), cabbage (0.25–6.89 mg/100 g), broccoli (0.10–3.96 mg/100 g), peas (2.32 mg/100 g), lettuce (1.29–1.65 mg/100 g), celery (1.68 mg/100 g), and pumpkin (0.62–2.82 mg/100 g) also contribute significantly to dietary lutein intake. Despite their high lutein content, bioavailability is relatively low due to the rigid structure of plant cell walls and protein binding. As a result, the efficiency of lutein absorption from raw vegetables can be limited unless processing is applied [70].

### 4.2. Fruits

In addition to green leafy vegetables, lutein is also present in a variety of fruits and other plant-based foods. Based on available data, fruits such as gooseberry (0.25–0.29 mg/100 g), peach (0.078 mg/100 g), kiwi (0.07 mg/100 g), apricot and banana (both ~0.03 mg/100 g), jackfruit (0.01–0.055 mg/100 g), and guava (0.003–0.011 mg/100 g) contain relatively small amounts of lutein [6]. Compared to vegetables, these values are significantly lower, indicating that fruits contribute modestly to overall dietary lutein intake on a per-mass basis. Among plant-based foods, maize (corn) is notable for its relatively higher lutein content (0.25–0.59 mg/100 g) and its importance as a staple crop in many regions [71]. Fruits like avocado offer an additional advantage due to their natural lipid content [72], which can enhance lutein absorption. However, overall, these sources provide less lutein than green vegetables on a per-mass basis.

### 4.3. Animal-Based Sources

Animal-derived foods, particularly egg yolk, represent important dietary sources of lutein with high bioavailability [73]. Available data indicate that egg yolk contains approximately 1.30–1.69 mg/100 g of lutein, which is considerably lower than the concentrations found in many green vegetables such as kale or spinach. Despite its lower absolute content, egg yolk is often regarded as one of the most effective dietary sources of lutein from a physiological perspective. This enhanced effectiveness is primarily attributed to its lipid-rich matrix, where lutein is naturally incorporated into lipid–protein complexes. Such a matrix facilitates efficient incorporation into mixed micelles during digestion, thereby significantly improving intestinal absorption [43]. In contrast to plant-based sources, lutein in egg yolk does not require extensive matrix disruption for release, making it more readily bioaccessible.

### 4.4. Limitations of Conventional Sources

Despite their nutritional importance, conventional lutein sources present several limitations. These include low concentration per biomass [74], meaning that large quantities of vegetables are required to meet recommended lutein intake levels. In addition, production is subject to seasonal and agricultural dependence [19], as climate, soil conditions, and farming practices influence it. There is also significant variability in lutein content, which can vary with cultivar, maturity stage, and post-harvest handling [75]. Furthermore, conventional cultivation is land- and resource-intensive, requiring substantial inputs of land, water, and other resources, thereby raising sustainability concerns.

## 5. Alternative and Emerging Sources of Lutein

The increasing demand for lutein in nutraceutical, pharmaceutical, and functional food industries has exposed the limitations of conventional plant-based sources. These include low productivity, seasonal variability, and high land and water requirements. As a result, research has shifted toward alternative and sustainable sources capable of providing higher yields, consistent quality, and scalable production. Among these, microalgae have emerged as one of the most promising platforms for lutein production [76]. Several alternative and emerging sources of lutein are summarized in Table 2.

### 5.1. Microalgae as a Primary Alternative Source

Microalgae are unicellular photosynthetic microorganisms recognized for their ability to synthesize a wide range of high-value compounds [106,107], including carotenoids such as lutein. Compared to terrestrial plants, microalgae offer several advantages, including higher growth rates [108], superior photosynthetic efficiency [109], and the ability to accumulate significant amounts of lutein under controlled conditions [19]. Several microalgal species have been identified as efficient lutein producers, including *Chlorella vulgaris* [110], *Chlorella sorokiniana* [111], *Scenedesmus almeriensis* [112], *Muriellopsis* sp. [92], and *Chlamydomonas* sp. [113]. Importantly, quantitative data demonstrate that microalgae can accumulate lutein at levels that are orders of magnitude higher than those in conventional plant sources. For instance, species such as *Acutodesmus* sp. (1744 mg/100 g) [77], *Chlorella sorokiniana* (up to 1730 mg/100 g) [74], and *Asterarcys quadricellulare* (1550 mg/100 g) [78] exhibit exceptionally high lutein content under optimized conditions. In contrast, conventional vegetables such as kale and spinach typically contain less than 40 mg/100 g, highlighting the substantial productivity advantage of microalgal systems. Even across different cultivation modes (photoautotrophic, mixotrophic, or heterotrophic), microalgae consistently achieve high lutein yields, underscoring their flexibility and industrial potential.

In microalgae, lutein biosynthesis occurs through the carotenoid metabolic pathway, which originates from the isoprenoid precursors isopentenyl pyrophosphate (IPP) and dimethylallyl pyrophosphate (DMAPP) [114]. These precursors are synthesized mainly through the methylerythritol phosphate (MEP) pathway in chloroplasts [115]. Subsequently, IPP and DMAPP are converted into geranylgeranyl pyrophosphate (GGPP), the key precursor for carotenoid biosynthesis [116]. The biosynthetic pathway proceeds through several enzymatic steps. Two molecules of GGPP are first condensed by *phytoene synthase* (*PSY*) to form phytoene [117]. Phytoene then undergoes sequential desaturation and isomerization reactions catalyzed by *phytoene desaturase* (*PDS*), *ζ-carotene desaturase* (*ZDS*), and *carotenoid isomerases* to produce lycopene [118]. Lycopene cyclization subsequently generates α-carotene through the coordinated activity of *lycopene ε-cyclase* (*LCYE*) and *lycopene β-cyclase* (*LCYB*) [119]. Finally, α-carotene is hydroxylated by *β-ring* and *ε-ring hydroxylases* to produce lutein [16].

Microalgae sources offer several key advantages as an alternative source of lutein. They exhibit high productivity by achieving rapid biomass accumulation, enabling continuous, high-yield lutein production [74]. In addition, their cultivation is non-competitive with agricultural land [19], as they can be grown in photobioreactors or open pond systems, reducing dependence on arable land and enabling production in non-agricultural regions. Microalgae for lutein production can be cultivated in either open-pond systems or closed photobioreactors, each offering distinct advantages and limitations [77,92]. Open pond systems are characterized by low operational costs and relatively easy scale-up, making them attractive for large-scale production [92]; however, they are more susceptible to contamination and environmental fluctuations, which can affect productivity and consistency. In contrast, closed photobioreactors provide greater control over growth conditions [93], resulting in improved productivity, product purity, and process stability, although they require higher capital investment and operational costs.

The productivity of microalgae-derived lutein is strongly influenced by cultivation regimes, as environmental and operational conditions directly affect both biomass accumulation and carotenoid biosynthesis. Different cultivation strategies, including photoautotrophic, heterotrophic, and mixotrophic cultivation, have been explored to optimize lutein production. In photoautotrophic cultivation, microalgae utilize light and CO_2_ as energy and carbon sources, making the process highly sustainable and environmentally attractive [15]. However, lutein accumulation under photoautotrophic conditions is highly dependent on light intensity, photoperiod, temperature, nutrient availability, and CO_2_ supply [19]. Moderate light intensities generally promote biomass growth, whereas high light stress can stimulate carotenoid accumulation as part of the cellular photoprotective response [120]. Nitrogen availability also plays a critical role, as moderate nitrogen limitation may enhance carotenoid synthesis [121], although severe nutrient stress often reduces biomass productivity.

Mixotrophic cultivation combines photosynthesis with the utilization of organic carbon sources, such as glucose or acetate, thereby enabling higher biomass productivity and faster growth rates than purely photoautotrophic systems [122]. This approach can improve lutein productivity because microalgae can simultaneously utilize light energy and external carbon substrates [123]. Heterotrophic cultivation, in contrast, relies entirely on organic carbon sources and eliminates dependence on light penetration, allowing high-cell-density fermentation. Nevertheless, heterotrophic cultivation is limited to specific strains capable of utilizing external carbon substrates and may increase production costs due to the need for additional substrates [123,124]. Two-stage cultivation strategies have also been widely investigated, where the first stage focuses on maximizing biomass growth under favorable conditions, followed by a stress-induction stage designed to enhance lutein accumulation through controlled exposure to high light intensity, altered nutrient conditions, salinity stress, or temperature shifts [125].

Furthermore, microalgae possess strong sustainability potential, as they can utilize CO_2_ as a carbon source and be integrated with wastewater treatment systems [125], contributing to carbon capture, resource recovery, and circular bioeconomy efforts. Yin and Miao [125] investigated the cultivation of *Chlorella sorokiniana* NIES-2168 using aquaculture wastewater supplemented with BG11 nutrients. Under optimized conditions with 2% CO_2_ aeration, the microalgae achieved a biomass concentration of 1.78 g/L and a lutein content of 7.43 mg/g. Furthermore, the implementation of a two-stage cultivation strategy increased lutein accumulation to 13.95 mg/g with a lutein productivity of 3.63 mg/L/day. Importantly, the system simultaneously removed 96.07% nitrate and 96.75% phosphate from the wastewater, demonstrating the dual benefits of lutein production and nutrient remediation. Similarly, Fariz-Salinas et al. [126] cultivated an autoflocculating microalgal consortium (BR-UANL-01) in secondary wastewater effluent from a treatment plant. The study reported lutein production of approximately 2.91 mg/g biomass under low-light conditions, while phosphorus removal efficiency exceeded 85%. In another study, Zheng et al. [111] used enzymatically pretreated corn starch wastewater to cultivate *Chlorella sorokiniana*. Using a cyclic feeding-cultivation strategy, the system achieved a lutein yield of 14.86 mg/L and a COD removal efficiency of 73.2%.

### 5.2. Agro-Industrial Byproducts

Agro-industrial byproducts have emerged as a cost-effective and sustainable alternative source of lutein, aligning closely with the principles of circular bioeconomy and waste valorization [127]. Large volumes of agricultural and food processing residues are generated globally, many of which still contain significant amounts of carotenoids, including lutein [128]. Instead of being discarded or underutilized, these byproducts can be converted into high-value bioactive compounds, offering both economic and environmental benefits.

Several agro-industrial residues have been identified as potential sources of lutein. Among these, marigold (*Tagetes erecta*) flower residues are the most established commercial source, widely used in dietary supplements and as natural food colorants; even after primary processing, the residual biomass still contains appreciable levels of lutein that can be further recovered [129]. Quantitative data indicate that marigold species can contain substantial lutein concentrations, ranging from approximately 21.6–97.6 mg/100 g in *Tagetes erecta* [4] to 59.7–123.1 mg/100 g in *Tagetes patula* [4], highlighting their strong potential as industrial feedstocks. Corn processing byproducts, such as corn gluten meal from starch and ethanol industries, also contain notable amounts of lutein and zeaxanthin [130]. For example, corn-derived byproducts have been reported to contain approximately 5.07–37 mg/100 g of lutein [95,96], depending on processing conditions, demonstrating their potential as secondary raw materials for extraction. Although it is commonly used in animal feed, it still offers potential for higher-value extraction. In addition, vegetable processing waste, including spinach stems, broccoli leaves, carrot peels, and green vegetables biomass, can serve as secondary sources of lutein [131]. Although the lutein content in these residues is generally lower than that of dedicated sources, their large volume and continuous availability make them attractive for bulk recovery within integrated biorefinery systems. Other plant residues, such as leaf waste generated during agricultural harvesting and trimming operations, may also contain recoverable amounts of lutein, although their concentrations can vary significantly depending on the source and conditions.

Utilizing agro-industrial byproducts for lutein extraction offers several key advantages. First, it enables significant cost reductions, as raw materials are considerably cheaper than dedicated crops or cultivated sources [132]. In addition, it contributes to waste minimization by reducing environmental pollution and landfill burden through the conversion of residues into value-added products [133]. This approach also supports sustainability and the integration of the circular economy by promoting resource efficiency and closing material loops within agro-industrial systems [134]. Furthermore, the continuous generation of byproducts ensures industrial availability, providing a relatively stable and scalable supply of raw materials for lutein production [135].

The recovery of lutein from agro-industrial byproducts requires efficient extraction strategies due to several inherent challenges. First, the complex biomass structure poses a significant barrier, as lutein is often embedded in plant tissues and bound to proteins or cellular membranes, necessitating pretreatment steps such as drying, grinding, or cell disruption to enhance its release [106]. In addition, conventional solvent-based extraction methods rely heavily on organic solvents, raising concerns about cost, safety, and environmental impact. To address these limitations, emerging green technologies, including supercritical CO_2_ extraction, ultrasound-assisted extraction, and enzyme-assisted extraction [136], are increasingly being explored to improve extraction efficiency while enhancing sustainability.

### 5.3. Bioengineered and Fermentation-Based Production

Bioengineered and fermentation-based systems represent an advanced, highly controllable approach to lutein production [137], offering an alternative to both agricultural and algal sources. By leveraging metabolic engineering and synthetic biology, microorganisms can be tailored to produce lutein efficiently under controlled conditions, independent of climate, seasonality, or land availability. This approach is increasingly gaining attention as part of next-generation biomanufacturing platforms for high-value carotenoids.

Native lutein production in microorganisms is generally limited [19]; therefore, genetic engineering is typically required to construct and optimize lutein biosynthetic pathways in suitable host organisms [137]. Several microbial platforms have been widely explored for this purpose. Bacteria such as *Escherichia coli* are frequently used due to their well-characterized genetics, rapid growth, and ease of genetic manipulation [138], allowing the introduction of lutein biosynthesis pathways through heterologous expression of carotenoid genes. However, current data indicate that bacterial systems often exhibit relatively low lutein yield, for example, around 11 mg/L and up to 218 mg/L in engineered *E. coli* [103,104], highlighting the need for further pathway optimization.

Yeasts, including *Saccharomyces cerevisiae* [139] and *Yarrowia lipolytica* [140], offer additional advantages in terms of robustness, scalability, and compatibility with established industrial fermentation processes. For instance, engineered *S. cerevisiae* has been reported to achieve lutein production levels of approximately 453 mg/L [105], demonstrating improved productivity compared to bacterial systems. Meanwhile, cyanobacteria and other photosynthetic microorganisms offer a hybrid approach by using CO_2_ and light, combining the features of microalgal systems with the flexibility of microbial engineering [141]. In addition, genetically modified microalgae represent an important intermediate platform, in which strains such as engineered *Chlamydomonas reinhardtii* and mutant *Chlorella zofingiensis* have demonstrated lutein contents ranging from 271 mg/100 g to as high as 1381 mg/100 g, indicating that genetic modification can significantly enhance lutein accumulation beyond wild-type levels. These engineered systems typically require the insertion and optimization of multiple genes involved in carotenoid biosynthesis, including pathways that convert isoprenoid precursors such as IPP and DMAPP into lutein [114].

To enhance lutein production, several metabolic engineering strategies are employed to optimize biosynthetic efficiency and maximize yield. One key approach is pathway reconstruction, which involves introducing complete lutein biosynthetic pathways from plants or microalgae into microbial hosts [137]. In addition, flux optimization is applied to increase the availability of key precursors, such as those derived from the mevalonate (MVA) or methylerythritol phosphate (MEP) pathways, thereby boosting overall carotenoid synthesis [142]. Further improvements can be achieved through gene overexpression and targeted knockouts, in which key enzymes in the lutein biosynthetic pathway are overexpressed, while competing metabolic pathways are suppressed or eliminated to redirect metabolic flux [143]. Moreover, promoter and regulatory engineering enable precise control of gene expression [144], allowing fine-tuning of pathway activity to enhance lutein accumulation.

Lutein production via fermentation typically involves several well-established cultivation strategies, with submerged fermentation (SmF) being the most commonly used approach [145]. This method allows precise control over key operational parameters such as pH, temperature, and oxygen levels, making it highly suitable for large-scale and consistent production. In addition, fed-batch and continuous fermentation systems are often employed to enhance productivity and improve substrate utilization [146]. These systems enable higher cell densities and increased lutein yields by maintaining optimal growth conditions over extended periods. Compared with photosynthetic systems, fermentation-based production offers shorter production cycles and greater scalability, as it is not dependent on light availability or environmental conditions [147]. However, it requires organic carbon sources, which can increase production costs and raise sustainability concerns if not sourced efficiently [148].

Bioengineered lutein production offers several important advantages that make it a promising alternative to conventional sources. One of the key benefits is its independence from environmental conditions [25], as production is not affected by climate, weather, or agricultural limitations, enabling year-round, location-independent manufacturing. In addition, it ensures high consistency and product quality [25] because controlled fermentation systems provide reproducible conditions and standardized outputs. Another major advantage is scalability, as bioengineered production can leverage well-established industrial fermentation technologies to achieve large-scale manufacturing [149]. Furthermore, there is strong potential for high yield, as advanced genetic and metabolic engineering strategies can significantly enhance lutein biosynthesis and accumulation within microbial hosts [137].

## 6. Extraction and Recovery Technologies

Efficient extraction and recovery of lutein are critical steps in determining its economic feasibility, purity, and functional quality. Given that lutein is typically embedded within complex biological matrices, extraction requires both cell disruption and selective solubilization [106]. Conventional techniques have been widely applied; however, growing environmental and economic concerns have driven the development of green and sustainable extraction technologies.

### 6.1. Conventional Solvent Extraction

Traditional extraction of lutein relies heavily on organic solvents such as hexane, acetone, ethanol, dichloromethane, and methanol [15,150], which are effective at dissolving lutein due to its lipophilic nature. Common techniques include maceration, Soxhlet extraction, and solvent partitioning [151,152]. These methods offer several advantages, including high extraction efficiency, simplicity, and well-established protocols that are applicable at both laboratory and industrial scales.

Table 2 indicates that solvent-based extraction remains the dominant method across a wide range of lutein sources, including microalgae [74], agro-industrial byproducts [95], and bioengineered microorganisms [97]. Most reported studies employ solvent systems such as acetone–methanol [79,102], ethanol [95], diethyl ether [87], or dichloromethane [81], often combined with pre-treatment steps such as saponification [91] or cell disruption [99] to enhance lutein recovery. For instance, high lutein yields in microalgae (often exceeding 1000 mg/100 g) are commonly achieved by solvent extraction coupled with saponification or mechanical disruption, highlighting the effectiveness of these methods for releasing intracellular carotenoids. Despite the high extracted lutein, they also present notable limitations. The high consumption of organic solvents increases operational costs and raises environmental and safety concerns, particularly due to their toxicity and flammability [153]. In addition, residual solvent contamination in the final product can pose quality and regulatory issues [154]. Solvent recovery processes are also considered energy-intensive [155].

### 6.2. Green Extraction Technologies

#### 6.2.1. Supercritical CO_2_ Extraction

Supercritical carbon dioxide (SC-CO_2_) extraction is one of the most promising green technologies for lutein recovery [156]. Under supercritical conditions (above 31 °C and 7.38 MPa), CO_2_ exhibits both gas-like diffusivity and liquid-like solvating power, enabling efficient penetration into biomass and selective solubilization of target compounds. This technique offers several advantages, including being non-toxic, non-flammable, and environmentally friendly, while leaving no solvent residues in the final product [156]. In addition, its selectivity can be tuned by adjusting pressure and temperature, making it suitable for high-purity applications in the nutraceutical and pharmaceutical industries.

Recent studies have demonstrated that SC-CO_2_ extraction is not only a green alternative to conventional solvent extraction but also an effective technique for the recovery of lutein from various biological matrices. Pal and Bhattacharjee [157] optimized SC-CO_2_ extraction of lutein from yellow maize kernels and reported a maximum lutein yield of approximately 275 μg/g dry weight at 500 bar and 70 °C after 90 min of extraction. Under these optimized conditions, the extract also exhibited the highest antioxidant activity and favorable phytochemical properties. The study further demonstrated that SC-CO_2_ extraction minimized thermal degradation and avoided residual organic solvent contamination, highlighting its suitability for nutraceutical-grade lutein production. Similarly, Di Sanzo et al. [158] investigated SC-CO_2_ extraction of lutein from *Haematococcus pluvialis* microalgae and achieved a lutein recovery of approximately 52.3% at 50 °C and 550 bar. The study showed that extraction efficiency was significantly influenced by pressure and biomass pretreatment, particularly mechanical cell disruption, which enhanced mass transfer and carotenoid release from the rigid microalgal cell wall. These findings emphasize that pretreatment strategies are critical for improving SC-CO_2_ extraction performance in microalgal systems. However, SC-CO_2_ extraction also has limitations. It requires substantial capital investment in specialized equipment and can be less efficient at extracting more polar compounds [159], often necessitating the use of co-solvents such as ethanol [156]. Furthermore, the process involves operational complexity, requiring precise control of pressure and temperature conditions.

#### 6.2.2. Ultrasound-Assisted Extraction (UAE)

Ultrasound-assisted extraction (UAE) utilizes acoustic cavitation, in which the formation, growth, and collapse of microbubbles generate localized shear forces that disrupt cellular structures and enhance mass transfer [160]. This process facilitates the release of lutein from biomass and improves its interaction with the extraction solvent. UAE offers several advantages, including reduced extraction time, lower solvent consumption, and improved extraction yield compared to conventional methods [161]. It is also considered relatively energy-efficient, making it an attractive option for sustainable processing. Saini and Panesar [162] optimized UAE for lutein extraction from kinnow peels and obtained a maximum lutein yield of 26.70 ± 2.00 μg/g under optimized conditions of a 6.40 mL/g solvent-to-solid ratio, 42.5 °C, a 34 min extraction time, and a 33% ultrasonic amplitude. The study showed that moderate temperatures and sonication intensity enhanced lutein diffusion from the plant matrix. Similarly, Liu et al. [163] reported that combining ultrasound with SC-CO_2_ extraction increased lutein yield from *Tropaeolum majus* flowers by approximately 14.9% while reducing extraction time by 16.7% compared to conventional SC-CO_2_ extraction. In addition, ultrasonic assistance improved mass transfer and enhanced lutein solubility in supercritical CO_2_, thereby increasing antioxidant and phytochemical yields under milder operating conditions. Despite its effectiveness, certain limitations must be considered. Prolonged ultrasound exposure may degrade lutein due to localized heat and free radical formation [162,164]. In addition, scaling up the UAE for industrial applications remains challenging because maintaining uniform energy distribution in larger systems is difficult [165].

## 7. Applications in Food and Health

### 7.1. Fortified and Functional Food Systems

Fortified and functional food systems represent a consumer-oriented approach to delivering lutein [25], enabling consumers to obtain health benefits through regular dietary intake [166]. Rather than focusing solely on lutein extraction and supplementation, this strategy emphasizes incorporating lutein into commonly consumed foods, thereby enhancing dietary intake in a convenient and accessible manner [9]. This approach is particularly relevant in addressing nutritional deficiencies and promoting preventive healthcare through everyday diets. Moreover, when delivered via lipid-containing matrices, such as dairy products, lutein bioavailability can be improved by enhanced micelle formation during digestion [38]. However, formulation challenges remain, particularly due to lutein’s poor water solubility and sensitivity to processing conditions, including heat, light, and oxygen [167].

Lutein can be incorporated into a wide range of food matrices to enhance their nutritional value and deliver health benefits through daily consumption. Common applications include dairy products (such as milk, yogurt, and cheese) [168], beverages (including fruit juices, smoothies, and functional drinks) [25], and bakery and cereal products [169]. In addition, lutein is increasingly utilized in specialized products such as infant formulas and medical nutrition formulations [170]. Fortification can be achieved either directly by adding purified lutein extracts, typically derived from marigold or microalgae, or by natural enrichment strategies [26], which involve incorporating lutein-rich ingredients into food formulations. These approaches enable the development of functional food products that support eye health and overall well-being while maintaining consumer convenience.

An alternative strategy to conventional fortification is biofortification, in which lutein content is enhanced during primary production rather than added during processing [25]. This approach can be implemented through both animal-based and crop-based systems. In animal-based biofortification, poultry are fed lutein-rich diets, such as those supplemented with marigold extract or microalgae, resulting in lutein-enriched egg yolks with improved bioavailability due to their lipid-rich matrix. In crop biofortification, plant breeding or genetic modification techniques are used to increase the intrinsic lutein content of crops.

### 7.2. Nutraceuticals

Lutein is widely used in the nutraceutical industry, particularly as dietary supplements designed to support eye health and overall well-being [25]. Common formulations include capsules and softgels, tablets, and powdered supplements [6]. These products are often combined with other synergistic compounds, such as zeaxanthin, omega-3 fatty acids, and vitamins A, C, and E, to enhance their efficacy [171]. Nutraceutical formulations provide a concentrated and standardized dose of lutein, making them especially suitable for individuals at risk of deficiency or those requiring targeted health support. The growing popularity of lutein-based nutraceuticals is driven by increasing awareness of digital eye strain and age-related vision disorders, the rising emphasis on preventive healthcare, and the continued expansion of the global dietary supplement market [172].

### 7.3. Pharmaceutical Applications

In the pharmaceutical sector, lutein is primarily utilized in formulations targeting ocular health, particularly for the prevention and management of vision-related disorders [9]. It is commonly included in clinically formulated eye supplements, for which its efficacy is supported by studies showing that it increases MPOD and reduces oxidative stress in retinal tissues [173]. Pharmaceutical-grade lutein products require high purity and stability, precise dosage control, and strict compliance with regulatory standards. In some cases, lutein is incorporated into combination therapies, working synergistically with other antioxidants and micronutrients to enhance therapeutic outcomes and improve overall efficacy [174].

## 8. Challenges and Research Gaps

**Figure 4:**
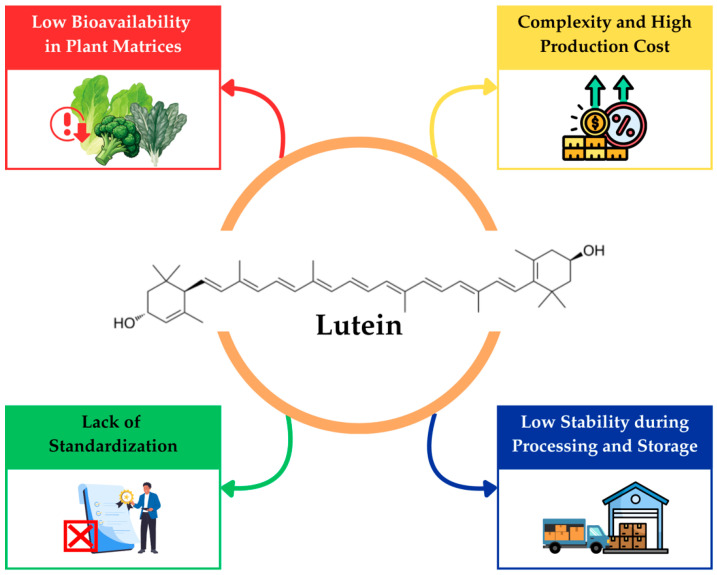
Challenge and Research Gaps in Lutein Production and Utilization.

Despite significant advancements in the production, extraction, and application of lutein, several critical challenges and research gaps continue to limit its full potential in food and health industries, as summarized in Figure 4 and detailed further in the following subsections.

### 8.1. Low Bioavailability in Plant Matrices

One of the major limitations of dietary lutein is its low bioavailability when derived from plant sources, particularly green vegetables [4]. Even foods with high lutein content may not necessarily translate into high physiological uptake. This underscores the need for improved processing techniques, such as homogenization and thermal treatment, to enhance lutein release, as well as the development of advanced delivery systems, such as emulsions and encapsulation. Additionally, more comparative studies are required to better understand lutein bioavailability across different food matrices and to optimize its nutritional efficacy.

Despite their promise, several challenges remain in the utilization of agro-industrial byproducts as lutein sources. One key issue is the variability in lutein content, as composition can differ significantly depending on plant type, seasonal factors, processing conditions, and storage [75]. In addition, some residues exhibit low lutein concentrations, requiring large volumes of biomass to achieve meaningful recovery, thereby reducing process efficiency.

### 8.2. Complexity and Production Cost

Microalgae are among the most promising alternative sources of lutein; however, their commercialization is hindered by high production and processing costs. Several upstream challenges continue to limit the large-scale commercialization of microalgae-based lutein production. One major issue is the high operational cost associated with cultivation systems, particularly closed photobioreactors that require continuous energy input for lighting, mixing, aeration, temperature control, and CO_2_ supply [175]. Although open pond systems offer lower costs, they are more vulnerable to contamination, evaporation losses, fluctuating environmental conditions, and inconsistent productivity [92]. Another upstream challenge involves light distribution and penetration in dense cultures, where self-shading effects can reduce photosynthetic efficiency and limit lutein biosynthesis [120]. Maintaining stable cultivation conditions at an industrial scale also remains technically challenging due to variations in temperature, nutrient availability, dissolved oxygen levels, and microbial contamination. Another major challenge is the complexity of lutein biosynthesis, which involves multiple enzymatic steps, making pathway construction and optimization technically demanding. Furthermore, achieving an optimal balance between biomass productivity and lutein accumulation remains challenging because stress conditions that stimulate carotenoid biosynthesis often suppress cellular growth.

Downstream processing also represents a major bottleneck in microalgae-based lutein production. Harvesting and dewatering of microalgal biomass are highly energy-intensive because microalgae are typically cultivated in dilute suspensions with low biomass concentrations [175]. Conventional harvesting techniques such as centrifugation, filtration, and flocculation can substantially increase operational costs. In addition, the rigid cell walls of many lutein-producing microalgae hinder efficient extraction [176], necessitating mechanical, chemical, or enzymatic cell disruption prior to solvent extraction [106]. Conventional solvent extraction methods also raise environmental and safety concerns due to high solvent consumption and the need for solvent recovery. Although emerging green extraction technologies such as supercritical CO_2_ and ultrasound-assisted extraction show promising potential, their industrial implementation is still constrained by equipment costs, process optimization challenges, and scalability limitations. Advanced formulation and encapsulation technologies might also increase product cost. In addition, regulatory and safety concerns surrounding the use of genetically modified organisms (GMOs) may pose obstacles, particularly in food-related applications [177], due to strict approval processes and varying levels of consumer acceptance. Current research gaps include the development of low-cost cultivation systems, the integration of microalgae production with wastewater treatment or CO_2_ capture to reduce operational costs, and the optimization of biorefinery approaches to enable the co-production of multiple valuable compounds, thereby improving overall process economics and sustainability.

### 8.3. Lack of Standardization

Another significant issue is the lack of standardization across studies and industrial practices, which complicates comparison, reproducibility, and regulatory approval. This challenge is reflected in the variability of reported lutein content due to differences in extraction and analytical methods, as well as inconsistent reporting of experimental conditions such as light intensity, solvent ratios, and processing parameters [19]. This lack of uniformity hinders effective meta-analysis and data comparison, limits industrial scalability and quality control, and creates barriers to regulatory acceptance in both food and pharmaceutical sectors.

### 8.4. Stability During Processing and Storage

Lutein is inherently unstable and prone to degradation, which poses significant challenges during processing, formulation, and storage. Its degradation is primarily driven by exposure to light, which induces photo-oxidation and isomerization; heat, which accelerates thermal degradation; and oxygen, which promotes oxidative breakdown [20,45]. These factors can result in a loss of bioactivity, reduced shelf life, and decreased overall product quality. Although various strategies, such as encapsulation and the addition of antioxidants, have been explored to improve lutein stability [46,178], further research is still required.

## 9. Future Perspectives

**Figure 5:**
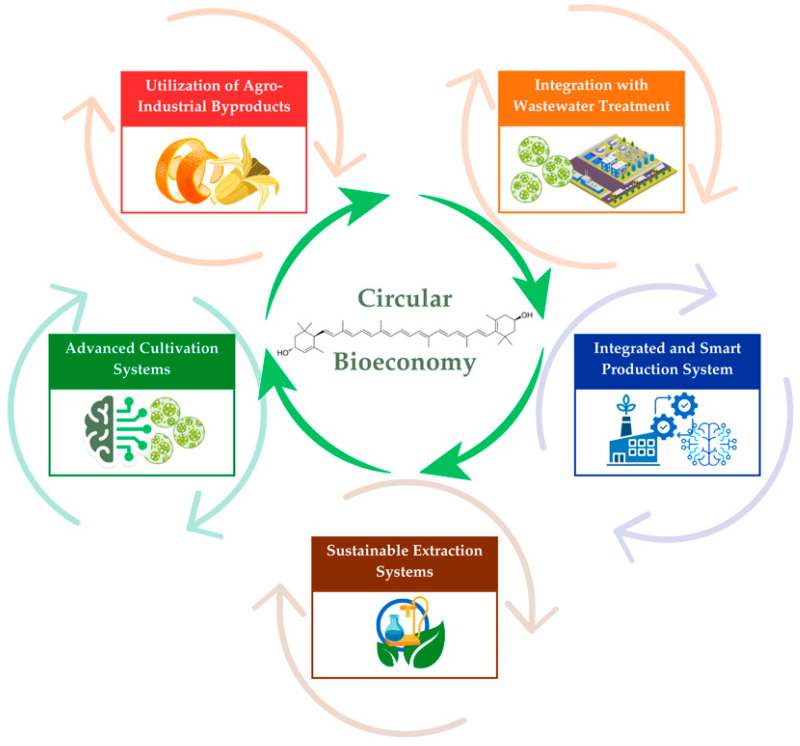
Future Framework for Sustainable Lutein Production.

Based on the challenges and gaps discussed in Section 8, future developments in lutein production are expected to focus on improving sustainability, process efficiency, scalability, and product stability through integrated, sustainable, and advanced technological approaches. The future directions and emerging strategies for sustainable lutein production are summarized in Figure 5.

### 9.1. Integration with Circular Bioeconomy

The concept of a circular bioeconomy emphasizes the efficient utilization of biological resources by converting waste streams into value-added products [127]. Within this framework (Figure 5), lutein production can be integrated into biorefinery systems, where multiple products are derived from a single biomass source to maximize resource efficiency. Future opportunities include using agro-industrial byproducts as low-cost feedstocks for lutein extraction [179] and integrating lutein production with wastewater treatment processes, particularly in microalgae cultivation systems [125].

The integration into the circular bioeconomy should also focus on the transition from laboratory scale to industrial scale in lutein production. One important strategy involves optimizing cultivation systems through improved photobioreactor and reactor design. Hybrid cultivation systems that combine open ponds with closed photobioreactors have also been explored to balance production costs and process control [180]. For fermentation-based systems, advanced bioreactor configurations with improved oxygen transfer, automated pH regulation, and real-time nutrient feeding strategies can enhance cell density and lutein productivity during large-scale operation [181]. Process intensification approaches are also increasingly important for industrial scale-up. Fed-batch and continuous cultivation systems can improve nutrient utilization, maintain stable growth conditions, and increase overall productivity compared to conventional batch processes [182,183]. In addition, two-stage cultivation strategies, in which biomass production and lutein induction are separated into distinct operational phases, have shown considerable promise for maximizing both biomass concentration and carotenoid accumulation [125]. Another critical strategy is to improve economic feasibility through biorefinery integration by co-producing lutein with other valuable compounds, such as proteins, lipids, and pigments, thereby significantly enhancing overall process economics [180].

### 9.2. Advanced and AI-Based Cultivation Optimization

Advances in synthetic biology, CRISPR-based genome editing, and systems biology modeling are expected to significantly enhance microbial lutein production [184] by enabling more precise and efficient pathway engineering. Moreover, hybrid approaches that combine metabolic engineering with biorefinery concepts have the potential to further optimize resource utilization and product yield [185].

In microalgae cultivation systems, artificial intelligence (AI) and machine learning (ML) can be applied to optimize critical parameters such as light intensity, photoperiod, temperature, pH, nutrient concentration, CO_2_ supply, and mixing conditions to maximize both biomass productivity and lutein accumulation [186]. Predictive models can also be used to monitor cellular stress responses and estimate lutein biosynthesis under varying environmental conditions [187]. Furthermore, AI-assisted smart sensor systems and automated control platforms can enable real-time monitoring and adaptive process control, thereby improving cultivation stability and reducing operational variability during large-scale production [187].

For fermentation-based lutein production, machine learning algorithms can assist in metabolic pathway optimization, strain selection, and prediction of fermentation performance under different operational strategies [188]. AI-driven metabolic modeling may also accelerate synthetic biology and genetic engineering efforts by identifying key regulatory bottlenecks and optimal gene expression patterns for enhanced lutein biosynthesis [189]. In downstream processing, AI tools can support optimization of extraction efficiency, solvent utilization, purification conditions, and encapsulation performance while minimizing energy consumption and processing costs. In addition, digital twin technologies integrated with AI may provide virtual simulation environments for process design, scale-up prediction, and industrial process troubleshooting [190]. Such approaches could substantially reduce experimental costs and improve the transition from laboratory-scale studies to commercial production systems.

### 9.3. Sustainable Extraction Systems

Future extraction technologies are expected to focus on minimizing environmental impact while maximizing efficiency and product quality. By coupling lutein extraction with the recovery of other valuable compounds [76], such as proteins, fibers, and polyphenols, the overall economic feasibility of agro-industrial byproducts into biorefinery frameworks can be significantly enhanced. Moreover, advances in green solvents and solvent-free extraction methods [191], as well as energy-efficient processes such as supercritical fluid extraction and ultrasound-assisted systems [192]. In addition, implementing closed-loop solvent recovery systems can significantly reduce chemical consumption and waste generation [155]. Hybrid extraction techniques that combine mechanical, enzymatic, and physical methods are anticipated to further enhance lutein recovery while preserving its stability [151]. Furthermore, future research should focus on establishing standardized extraction and analytical protocols, developing reference materials and benchmarking systems, and improving transparency and consistency in scientific reporting.

### 9.4. Integrated and Smart Production Systems

A key emerging trend is the development of integrated production platforms that combine cultivation, extraction, and formulation into a unified system [193]. These systems may incorporate smart sensors and automation, AI-driven decision-making tools, and continuous processing technologies to enable more efficient and controlled operations. Future efforts should also focus on developing more robust stabilization techniques, gaining a deeper understanding of degradation kinetics across different processing conditions, and designing effective protective delivery systems compatible with diverse food matrices.

## 10. Conclusions

Lutein is a highly valuable xanthophyll carotenoid with well-established roles in ocular protection, antioxidant defense, and overall human health. Its increasing demand in functional foods, nutraceuticals, and pharmaceutical applications reflects a broader shift toward preventive healthcare and bioactive-rich diets. However, reliance on conventional dietary sources such as leafy vegetables is limited by low bioavailability, variable content, and resource-intensive production, necessitating the exploration of more efficient and sustainable alternatives. This review highlights that lutein is a high-value carotenoid with significant applications in food, nutraceutical, pharmaceutical, and functional health sectors due to its antioxidant, anti-inflammatory, neuroprotective, and ocular protective properties. Conventional lutein sources, particularly green vegetables and marigold flowers, remain important; however, their limitations in bioavailability, seasonal dependency, and sustainability have accelerated the development of alternative production platforms. Among the emerging approaches, microalgae such as *Scenedesmus almeriensis*, *Muriellopsis* sp., and *Chlorella sorokiniana* demonstrate particularly strong potential due to their high lutein productivity, rapid growth, and ability to be cultivated under controlled conditions. Agro-industrial byproducts and bioengineered microbial systems also represent promising sustainable alternatives that align with circular bioeconomy principles and waste valorization strategies. This review further demonstrates that advances in green extraction technologies, including supercritical CO_2_ extraction and ultrasound-assisted extraction, can improve lutein recovery while reducing environmental impact compared to conventional solvent-based methods.

Despite these advancements, several challenges remain, including low bioavailability in plant matrices, compound instability, high cultivation and downstream processing costs, extraction efficiency, standardization issues, and industrial-scale implementation. Overcoming these barriers will require the integrated optimization of both upstream cultivation and downstream processing systems through innovations in biotechnology, process engineering, and materials science. The future of sustainable lutein production will depend on integrating advanced bioprocess engineering, AI-assisted optimization, green extraction technologies, and biorefinery concepts to achieve economically viable, scalable, and environmentally sustainable production systems that meet the growing global demand for natural lutein products.

## Figures and Tables

**Table 1:** Conventional Dietary Sources of Lutein and Its Concentration.

Category	Source	Lutein Content (mg/100 g)	Reference
Vegetables	Broccoli	0.10–3.96	[4,63,64]
	Cabbage	0.25–6.89	[63,64]
	Carrot	0.36	[64]
	Celery	1.68	[63]
	Cilantro	7.7	[4]
	Kale	14.7–39.6	[65]
	Lettuce	1.29–1.65	[4,63]
	Maize	0.25–0.59	[4,64]
	Peas	2.32	[63]
	Pumpkin	0.62–2.82	[4,63]
	Spinach	6.26–8.95	[63,66]
Fruits	Apricot	0.03	[64]
	Banana	0.03	[64]
	Gooseberry	0.25–0.29	[4]
	Guava	0.003–0.011	[4]
	Jackfruit	0.01–0.055	[4]
	Kiwi	0.07	[64]
	Peach	0.078	[64]
Animal-based	Egg yolk	1.30–1.69	[67,68]

**Table 2:** Comparison of Alternative Lutein Production Sources.

Source	Species	Detail	Extraction	Lutein Content (mg/100 g)	Reference
Microalgae	*Acutodesmus* sp.	Medium: BG-11 Temperature: 25 ± 1 °C Light intensity: 30 μmol photons/m^2^s Aeration: Continuous supply at 0.1 volume of air per volume of medium per minute (vvm) pH: 7.5 Reactor: Photobioreactors	Saponification-based extraction	1744	[77]
	*Asterarcys quadricellulare* PUMCC 5.1.1	Medium: BG-11 Temperature: 28 ± 2 °C Light intensity: 40 μmol photons/m^2^s Light regime: 14 h light/10 h dark Initial condition: OD_720_ ≈ 0.1 Reactor: 250 mL Erlenmeyer flasks (100 mL working volume)	Solvent extraction (petroleum ether: cyclohexane: ethyl acetate: acetone: methanol (60:16:10:10:4, *v*/*v*)	1550	[78]
	*Auxenochlorella protothecoides*	Medium: Basal medium (+5 g/L glycine for photoautotrophic and +30 g/L glucose + 0.5 g/L glycine for heterotrophic) Temperature: 28 ± 1 °C Light intensity: Photoautotrophic: 2000 lux Transition stage: 4000 lux Agitation: 220 rpm Reactor: 100 mL flask	Solvent extraction (acetone: methanol (8:2, *v*/*v*))	499	[79]
	*Chlamydomonas* sp. JSC4	Medium: BG-11 + 2% sea salt (preculture)/Modified Bold 3N medium (main cultivation) Temperature: Preculture: 28 °C Cultivation range: 20–45 °C (optimal growth at 35 °C) Light intensity: 250 μmol photons/m^2^s Aeration: Continuous supply of 2.5% CO_2_ at 0.15 vvm pH: 7.5 Agitation: 500 rpm Reactor: 1 L glass photobioreactor (LED)	Solvent extraction (diethyl ether)	424	[80]
	*Chlorella protothecoides*	Medium: Basal medium (glucose 30 g/L as control) and Monascus fermentation broth medium (diluted 1:2) Temperature: 28 °C Light intensity: heterotrophic Reactor: Shake flasks (250 mL, 100 mL working volume) Agitation: 160 rpm Fed-batch: 1 L bioreactor	Solvent extraction (methanol: dichloromethane (3:1, *v*/*v*)) + Saponification	911	[81]
	*Chlorella salina*	Medium: Walne’s medium Temperature: 24 to 28 °C Light intensity: 100–400 μmol photons/m^2^s Aeration: 1–5 L/min pH: 8.0 Reactor: Airlift photobioreactor (8 L, cylindrical, externally illuminated)	Solvent extraction (80% acetone)	1015	[82]
	*Chlorella sorokiniana* F31	Medium: BG-11 (+800 mg/L NaNO_3_) Temperature: 30 °C Light intensity: 100–284 μmol photons/m^2^s Aeration: Continuous 2.5% CO_2_ at 0.2 vvm pH: 7.5 Agitation: 300 rpm Reactor: 1 L photobioreactor (LED)	Solvent extraction (diethyl ether) + Saponification	1555	[83]
	*Chlorella sorokiniana* FZU60	Medium: BG-11 (0.75 g/L NaNO_3_ + 1 g/L sodium acetate) Temperature: 33 °C Light intensity:150–750 μmol photons/m^2^s Aeration: Continuous 2.5% CO_2_ at 0.15 vvm pH: 8.0 Agitation: 400 rpm Reactor: 1 L photobioreactor	Solvent extraction (ethyl ether)	1122	[84]
	*Chlorella sorokiniana* FZU60	Medium: Modified Mann and Myers medium (with glucose 10 g/L + nitrate/urea + trace elements) Temperature: 30 °C Light intensity: heterotrophic Aeration: 1 vvm pH: 7.5 Reactor: Shake flask (250 mL, 100 mL working volume) Agitation: 200 rpm Reactor: 5 L bioreactor	Solvent extraction (ethyl ether)	1381	[85]
	*Chlorella sorokiniana* FZU60	Medium: BG-11 (with sodium acetate + NaNO_3_ optimization) Temperature: 33 °C Light intensity: 150 μmol photons/m^2^s Aeration: 2.5% CO_2_ at 0.15 vvm Agitation: 300 rpm Reactor: 1 L glass photobioreactor	Solvent extraction (ethyl ether)	957	[86]
	*Chlorella sorokiniana* Kh12	Medium: TAP (Tris Acetate Phosphate) medium Temperature: 26–32 °C Light intensity: 10 k lux Aeration: Continuous 5% CO_2_ at 25 cc/min (microbubble aeration) Agitation: Air-driven mixing Reactor: 1 L bubble column photobioreactor (800 mL working volume)	Solvent extraction (methanol: dichloromethane = 2.5:1, *v*/*v*)	1730	[74]
	*Chlorella sorokiniana* MB-1-M12	Medium: BG-11 + 6 g/L sodium acetate (mixotrophic) Temperature: 25 °C Light intensity: 150 μmol photons/m^2^s Aeration: Continuous 2% CO_2_ at 0.1 vvm Agitation: 300 rpm Reactor: 60 L tubular photobioreactor (outdoor)	Solvent extraction (diethyl ether) + saponification	648	[87]
	*Chlorella sorokiniana* MB-1-M12	Medium: BG-11 + 7.5 g/L glucose + 0.75 g/L urea Temperature: 25 °C Light intensity: heterotrophic Aeration: 0.3 vvm pH: 7.5 Agitation: 300 rpm Reactor: 1 L glass photobioreactor	Solvent extraction (diethyl ether) + saponification	210	[21]
	*Chlorella* sp. AE10	Medium: BG-11 Temperature: 28 ± 0.05 °C Light intensity: 850 μmol photons/m^2^s Aeration: 20% CO_2_ at 0.2 L/min Reactor: Bubble column photobioreactor (350 mL working volume)	Solvent extraction (dichloromethane: methanol) + bead disruption	958	[88]
	*Chlorella vulgaris* CS-41	Medium: MLA (with nitrate-enriched variation) Temperature: 26–28 °C Light intensity: 160–760 μmol photons/m^2^s Aeration: 0.2–1 vvm air + CO_2_ supplementation Reactor: Photobioreactor (5 L flat-panel; 50 L bubble column)	Solvent extraction (Methyl tert-butyl ether) + bead homogenization	1055	[89]
	*Desmodesmus protuberans*	Medium: WC medium Temperature: 23 ± 1 °C Light intensity: 200 μmol photons/m^2^s Aeration: Air + 4% CO_2_ at 0.1 L/min pH: 7.0 Agitation: Air bubbling Reactor: Glass cylindrical bottles (1.8 L)	Solvent extraction (hexane: ethanol) + ultrasonic assistance	1053	[90]
	*Desmodesmus* sp. F51	Medium: Modified Bristol’s medium (preculture in BG-11) Temperature: 30 °C Light intensity: 150 μmol photons/m^2^s Aeration: CO_2_ (0.03–12.5%) at 0.2 vvm pH: 7.5 Agitation: 300 rpm Reactor: 1 L glass photobioreactor	Solvent extraction (diethyl ether) + saponification + bead disruption	556	[91]
	*Muriellopsis* sp. (MCH35)	Medium: UMA5 medium (adapted to seawater conditions) Temperature: 16.3–19.6 °C Light intensity: natural sunlight Aeration: Continuous aeration 0.1 vvm pH: 7.9–8.2 Reactor: Open raceway ponds (36 m^2^, 5.4 m^3^)	Supercritical fluid extraction (SFE) with CO_2_ + ethanol	420	[92]
	*Parachlorella* sp. JD-076	Medium: BG-11 Temperature: 35 °C Light intensity: 100–1000 μmol photons/m^2^s Aeration: 5% CO_2_ at 0.5 vvm Reactor: Tubular photobioreactor (7 L working volume)	Solvent extraction (ethanol) + bead disruption	1187	[93]
	*Scenedesmus almeriensis*	Medium: Modified Mann and Myers medium Temperature: 28 °C Light intensity: 4000 lux (LED) Aeration: Gas mixture (O_2_ + N_2_ + CO_2_) Flow rate: 300 mL/min (0.01 vvm) CO_2_ concentration: 0–3% pH: 7.5–8.5 Reactor: Vertical bubble column photobioreactor (28.5 L)	Accelerated solvent extraction (ethanol)	854	[94]
Agro-industrial byproducts	Corn Processing By-products	Raw material: Corn gluten meal (by-product of corn wet milling) Processing stage: By-product from starch production	Solvent extraction (ethanol)	20–37	[95]
	Corn Processing By-products	Raw material: Corn (*Zea mays* L.) Pre-treatment: Steeping (53 °C, 24 h, acidic solution) Processing method: Wet milling + mechanical separation	Solvent extraction (Acetone: petroleum ether (1:1, *v*/*v*))	5.07	[96]
	Marigold (*Calendula officinalis)*	-	Solvent extraction	4–30	[4]
	Marigold (*Tagetes erecta*)	-	Solvent extraction	21.6–97.6	[4]
	Marigold (*Tagetes patula)*	-	Solvent extraction	59.7–123.1	[4]
	White Bryony	-	Solvent extraction	19.13	[4]
Bio-engineered microorganisms	*Chlamydomonas reinhardtii*	Method: Gene manipulation Medium: High-salt (HS) medium Temperature: 25 °C Light intensity: 200 μmol photons/m^2^s Aeration: 5% CO_2_ bubbling (80 mL/min) Reactor: Bubble column photobioreactor (400 mL)	Solvent extraction (acetone)	271–308	[97]
	*Chlamydomonas reinhardtii*	Method: Lycopene epsilon-cyclase gene from *Chlorella vulgaris* Medium: glucose (10 g/L), yeast extract (1 g/L), peptone (1 g/L), tryptone (2 g/L), FeSO_4_·7H_2_O (2 mg/L) Temperature: 28 °C Light intensity: heterotrophic Reactor: lab-scale flask cultivation	Solvent extraction (acetone: dichloromethane) + freeze-drying)	2.3-fold higher than wild type	[98]
	*Chlamydomonas reinhardtii*	Method: Phytoene-β-carotene synthase gene from the red yeast *Xanthophyllomyces dendrorhous* Medium: TAP (Tris-acetate-phosphate) medium Temperature: 25 °C Light intensity: 75–900 μmol photons/m^2^s Reactor: lab-scale flask cultivation	Solvent extraction (methanol) + liquid nitrogen cell disruption	890	[99]
	*Chlamydomonas reinhardtii*	Method: CRISPR−Cas9 RNP Medium: modified 1/4NP2A medium (reduced N & P, higher acetate) Temperature: 25 °C Light intensity: 60 μmol photons/m^2^s Reactor: lab-scale flask cultivation	Solvent extraction (food-grade hexane/isopropanol) + ultrasound-assisted extraction	293	[100]
	*Chlorella sorokiniana* MB-1-M12 mutant	Method: Random mutagenesis Medium: BG-11 + 6 g/L sodium acetate (mixotrophic) Temperature: 25 °C Light intensity: 150 μmol photons/m^2^s Aeration: Continuous 2% CO_2_ at 0.1 vvm Agitation: 300 rpm Reactor: 60 L tubular photobioreactor (outdoor)	Solvent extraction (diethyl ether) + saponification	752	[87]
	*Chlorella vulgaris* NRF 13	Method: Random mutagenesis Medium: Nitrogen-limited BG-11 (NaNO_3_ reduced to 0.8 mM) Temperature: 24–25 °C Light intensity: 70–2500 μmol photons/m^2^s Aeration: Air + CO_2_ mixture pH: 6.8–7.2 Reactor: Photobioreactor (1 L, semi-batch system)	Solvent extraction (dimethylformamide)	5.4-fold higher than wild type	[101]
	*Chlorella zofingiensis* mutant (CZ-bkt1)	Method: Chemical mutation eliminating *BKT1* Medium: Kuhl medium with glucose supplementation Temperature: 25 °C Light intensity: 30–460 μmol photons/m^2^s pH: 7.0 Reactor: lab-scale flask cultivation	Solvent extraction (acetone)	1381	[102]
	*Escherichia coli*	Method: Lycopene ε-, β-cyclase and cytochrome P450 CYP97C encoding Medium: Modified Terrific Broth (TB) medium Temperature: 25 °C Light intensity: heterotrophic bacterial system Aeration: 1 vvm pH: 7.0 Reactor: 3 L jar fermenter	Solvent extraction (methanol: chloroform)	11 mg/L	[103]
	*Escherichia coli*	Method: *Photorhabdus luminescens* CipB scaffold protein channeling Medium: R/2 medium + glycerol (20 g/L) + yeast extract Temperature: 28–30 °C Light intensity: heterotrophic bacterial system Aeration: Shaking (200–220 rpm) pH: 6.8 Reactor: lab-scale flask cultivation and Fed-batch bioreactor	-	218 mg/L	[104]
	*Saccharomyces cerevisiae*	Method: δ-carotene formation and conversion genes insertion Medium: Glucose-based medium Light intensity: heterotrophic yeast system Reactor: lab-scale flask cultivation	-	453	[105]

## Data Availability

No new data were created or analyzed in this study. Data sharing is not applicable to this article.
